# Microbial flora of the respiratory tract and skin of artisanal municipal solid waste handlers in Aba, Abia State, Nigeria

**DOI:** 10.1099/acmi.0.000876.v5

**Published:** 2025-01-21

**Authors:** I.W. Nwankwo, N.C. Nwachukwu, E.C. Onwuchekwa, O.C. Okamgba, O.C. Ugbogu

**Affiliations:** 1 Department of Microbiology, Abia State University, Uturu, Nigeria; 2 Department of Medical Laboratory Science, Abia State University, Uturu, Nigeria; 3 Department of Biological Sciences, Amadeus University Amizi, Abia State, Nigeria

**Keywords:** artisanal waste handlers, microbial flora, municipal solid waste, Nigeria, respiratory tract, skin

## Abstract

Municipal solid waste handling carries occupational risk for waste handlers due to exposure to diverse microorganisms and hazardous substances that cause respiratory and skin infections. A cross-sectional study was carried out, and 150 respondents were recruited using a simple random sampling technique. The sociodemographic characteristics, health-related complaints, health-seeking behaviour and the bacterial and fungal microflora of the respiratory tract and skin of artisanal municipal solid waste handlers and some controls in Aba, Nigeria, were determined using a mixed methods research design, involving the use of interviewer-administered structured questionnaires and conventional culture techniques. We analysed the data using IBM SPSS version 25. The results are presented in tables as frequencies and percentages. The majority of artisanal municipal solid waste handlers in Aba are male (95%). Their mean age is 30 years, and the age group of 21–40 years constitutes the highest proportion (65%) of the workforce. Health-related complaints were higher (95%) among the waste handlers compared to the control subjects (4%). The findings show a high prevalence of respiratory (57%), eye (34%) and skin (87%) complaints among the waste workers, compared to 6%, 0% and 2%, respectively, among the control subjects. Seventy-eight (78%) of the waste handlers indulge in self-medication via over-the-counter (OTC) drugs; 17 (17%) access diagnostic laboratories and only 4 (4%) visit hospitals for treatment, as compared to the control subjects, who recorded 1 (2%) for OTC drugs, 46 (92%) for laboratories and 2 (4 %) for hospitals. Acquisition (15%) and use (3%) of personal protective equipment (PPE) were very low amongst the waste handlers. A total of 704 bacterial isolates and 191 fungal organisms were isolated from the study subjects. Among the waste handlers, the percentage distribution of bacteria was almost the same at both sites: respiratory tract, 241 (49.9%) and skin, 242 (50.1%), compared to the control subjects’ respiratory tract, 105 (47.5%) and skin, 116 (52.5%), which showed a slight difference between the sites. *S. epidermidis* (24%) and *B. cereus* (13%) were predominant in the respiratory tract, whereas *S. aureus (29%) and S. epidermidis* (19%) predominated the skin of the waste handlers. Similarly, *S. aureus* (34.3%) and *B. cereus* (20.9%) were predominant in the respiratory tract, while *B. cereus* (37.9%) and *S. epidermidis* (18.1%) predominated the skin of the control subjects. *Candida* spp. was the most predominant fungus in the respiratory tract (81.4%) and skin (42.9%) of the waste handlers, as well as in the respiratory tract (85%) and skin (78%) of the controls. The presence of the isolated bacteria and fungi in increased proportions in the waste handlers may be attributed to occupational exposure through direct contact with waste, inhalation of organic dust laden with biological agents and the poor working conditions of the waste handlers. Health education and improvements in working conditions are necessary to mitigate the occupational challenges of waste handlers.

## Data Summary

The data obtained for this study cover the sociodemographic characteristics, health-related complaints, health-seeking behaviour and bacterial and fungal microflora of the respiratory tract and skin of artisanal municipal solid waste handlers and some controls in Aba, Nigeria. The authors confirm that relevant data have been provided within the article, and the raw data used for this article is available on Figshare: https://doi.org/10.6084/m9.figshare.26125489.v1 [1].

## Introduction

Municipal solid waste (MSW) handling and disposal is a growing environmental and public health concern in many countries, including Nigeria. This is due to the high volume of waste generated within the municipality and inefficient management practices, which pose risks for the waste handlers, the environment and the populace. Waste management, which comprises collection, transport, sorting, processing and disposal, is a hard job [2,3]. Waste management carries a substantial occupational risk to its handlers [4]. In developing countries, waste collection activities are carried out in micro- and small-scale enterprises with old equipment and virtually no dust control or worker protection. The waste handler’s job involves tedious motion, uncomfortable working positions, deadly hand exertion and manual handling and constant daily activities without rest [5].

Solid waste handlers are prone to health-affecting conditions like cough, asthma, throat dryness, nasal discharge, skin diseases, breathing difficulties, etc. because of exposure to infectious microorganisms (bacteria, fungi, viruses, parasites and cysts), toxic substances (endotoxins, exotoxins and beta-glucans), chemicals that come from waste, bioaerosols generated by decaying organic matter and microorganisms, vehicle exhaust and fumes [3,9]. Waste handling activities bring the waste handler into contact with aerosolised bacteria and their bioproducts in the form of bioaerosols. Bioaerosols have been recognised to cause diseases among waste handlers and residents in the vicinity of waste processing facilities [10]. These biologically active entities, which carry pathogens and allergens, are transmitted via the air and penetrate the body through the nasal and oral mucosa and the skin [11]. Bacteria present in the respiratory tract and on the skin of the waste handlers could be responsible for various respiratory and skin conditions. In addition to exposure by inhalation, other exposure routes, such as ingestion and skin contact, present additional exposure risks to the workers [12]. Ivens *et al*. [13] posit that exposure to bacterial endotoxins or fungi carries a dose-response relationship with nausea and diarrhoea, while a similar relationship was found between exposure to bacteria and acute nasal irritation [14]. Waste handling work has been linked to symptoms and inflammation of the airways such as allergic rhinitis and allergic bronchopulmonary aspergillosis in waste handlers [15].

Municipal solid waste workers are constantly in contact with hazardous substances and infectious microorganisms (such as bacteria and fungi) found in waste due to occupational exposure. This puts them at risk of diverse ailments. Therefore, this study was carried out to isolate and characterise the bacteria and fungi constituting the microbial flora of the respiratory tract and skin of artisanal municipal solid waste handlers in Aba, Abia State, and their controls.

## Methods

### Study design and sampling

This cross-sectional study employed a mixed-methods research design. One hundred and fifty respondents were recruited – 100 private waste handlers and 50 student volunteers, who served as control subjects. Private artisanal municipal solid waste handlers who had been on the job for a minimum of 6 months were recruited into the study using a simple random sampling technique by the balloting method, until the desired number of subjects was obtained from 10 dumpsites across the municipality. An interviewer-administered structured questionnaire was used to obtain relevant information from the respondents. Data collected were analysed at the univariate and bivariate levels, employing appropriate statistics using IBM SPSS version 25. The per cent incidence was determined by dividing the total frequencies of bacterial and fungal isolates from each sample site by the total number of isolates from both sites and multiplied by 100% for the respective study groups. Results were presented as frequencies and percentages.

### Specimen collection, culture and identification of bacterial and fungal isolates

Nasal swabs were collected from the external nares of the nostrils by using a sterile cotton swab moistened with distilled water. The swab was placed in the nasal cavity, and the nostril was pressed from the outside against the swab with the index finger, and the swab was slightly rotated for maximum contact; this was done in both nostrils. Also, the skin swabs were collected using a sterile cotton swab moistened with distilled water and swept back and forth across the zone 1 portion of the neck, from the left supraclavicular fossa to the right from the subjects for culture and isolation of bacteria and fungi. The swab sticks were transported back to the laboratory in peptone water and incubated in the same for 4 h in a laboratory incubator at 37 °C before culturing in the respective bacteria and fungi culture media. The media used for organism enrichment was peptone water (TM 330, TM Media, Titan Biotech Ltd., Rajasthan, India). MacConkey agar (M1582, Himedia Laboratories Pvt Ltd., India), Mannitol salt agar (MH118, Himedia Laboratories Pvt Ltd., India) and Chocolate agar (M001, Himedia Laboratories Pvt Ltd., India) were used for bacterial isolation, and Sabouraud dextrose agar (SDA) (MH063, Himedia Laboratories Pvt Ltd., India) was used for fungi isolation. They were prepared according to the manufacturers’ instructions and autoclaved at 121 °C for 15 min. After incubation in peptone water, the nasal and skin swab specimens were cultured for bacteria isolation by the streak plate method [16,17]. A flame-sterilised wire loop was used to collect a loop-full of each of the specimens and was streaked on solid agar media (Mannitol salt agar, MacConkey agar and lysed blood agar) in 100 mm x 15 mm Petri dishes by the streak plate method and incubated for 24–48 h at 37 °C in a laboratory incubator (DNP-9052A, Zenith Lab., China).

For fungal isolation, the respective specimens were plated on SDA media by the streak plate method [16,17]. A flame-sterilised wire loop was used to collect a loop-full of each nasal swab and skin swab, which were streaked on the surface of the SDA in 100 mm x 15 mm Petri dishes and incubated at room temperature for 3–7 days to obtain fungal growth.

Bacterial isolates were characterised and identified using cultural characteristics, and phenotypic features of the bacterial isolates, Gram staining with microscopic visualisation for Gram reaction, cell shape and arrangement were used for characterisation of the isolates. Biochemical identification and carbohydrate utilisation tests such as catalase, coagulase, oxidase, indole, citrate, urease, methyl red, Voges-Proskauer, glucose, sucrose, lactose, maltose, mannitol and xylose were employed to identify bacterial isolates by their enzymatic and fermentation reactions, following the biochemical identification protocol [18,20].

Fungi isolates were identified based on their macroscopic and microscopic appearances [21]. The macroscopic features utilised included surface texture, surface topography, surface pigmentation, reverse pigmentation and growth rate, while the microscopic identification was based on the visualisation of wet preparation of the isolates using lactophenol cotton blue stain. The morphology and nature of spores and conidia served important diagnostic purposes in identification. The observed microscopic structures were compared with the presentations of Kidd *et al*. [21].

## Result

The frequency distribution of the sociodemographic characteristics of the waste handlers and the control subjects is shown in Table 1. Among the waste handlers, 95% were male, whereas among the control subjects, 68% were female and 32% were male. For age, the category with the highest proportion amongst the waste handlers was the 21–30 (46%) age group, as opposed to the 21–30 (64%) age group for the control subjects. For marital status, among the waste handlers, the majority were single (71%), and this is comparable to the control subjects (80%).

**Table 1.** Some sociodemographic characteristics of the waste handlers and the control subjects

**Sociodemographiccharacteristics**	**Waste handlers**		**Control subjects**	
	**freq.**	**(%)**	**freq.**	**(%)**
Sex:				
Male	95	(95)	16	(32)
Female	5	(5)	34	(68)
Age groups (years):				
11–20	16	(16)	9	(18)
21–30	46	(46)	32	(64)
31–40	19	(19)	7	(14)
41–50	14	(14)	2	(4)
51–60	3	(3)	0	(0)
61+	1	(1)	0	(0)
Marital status:				
Single	71	(71)	40	(80)
Married	29	(21)	10	(20)
Highest level of education:				
None	2	(2)	0	(0)
Primary	33	(33)	0	(0)
Secondary	62	(62)	10	(20)
Diploma	0	(0)	1	(2)
Polytechnic	0	(0)	1	(2)
Coll. Of Health Tech. (tertiary)	1	(1)	35	(70)
University	2	(2)	3	(6)

Regarding the level of education, among the waste handlers, 62% attained secondary school, while 33% had primary; only 2% had tertiary level education and no education, respectively.

Chi-square cross-tabulation shows the relationship between study subjects’ occupation and health complaints, use of personal protective equipment (PPE) and health-seeking behaviour (shown in Table 2).

**Table 2.** Association between subjects’ occupation and some dependent variables

Factors	Waste handlers		Control subjects		Chi-square	p- value
	Yes (%)	Total	Yes (%)	Total		
Health complaints	95 (95)	100	2 (4)	50	120.808	0.000
Cough	70 (70)	100	1 (2)	50	61.829	0.000
Diff. Breathing	48 (48)	100	0 (0)	50	35.294	0.000
Chest pain	26 (26)	100	0 (0)	50	15.726	0.000
Bloody cough	1 (1)	100	0 (0)	50	0.503	0.478
Nasal congest	80 (80)	100	2 (4)	50	77.690	0.000
Runny nose	61 (61)	100	0 (0)	50	51.404	0.000
Itchy eyes	34 (34)	100	0 (0)	50	21.983	0.000
Skin rashes	72 (72)	100	0 (0)	50	69.231	0.000
Itchy skin	87 (87)	100	0 (0)	50	103.571	0.000
Discol. Skin	95 (95)	100	1 (2)	50	125.130	0.000
Thickened skin	92 (92)	100	0 (0)	50	118.966	0.000
Ever admitted	6 (6)	100	0 (0)	50	3.125	0.077
Adm. Past yr.	4 (4)	100	0 (0)	50	2.055	0.152
Ever had lab test since on this job	26 (26)	100	4 (8)	50	6.750	0.009
Lab test past yr.	12 (12)	100	5 (10)	50	0.133	0.716
Have PPE	15 (15)	100	na			
Use PPE	3 (3)	100	na			
Source of treatment:	Hosp.4 (4), lab.17 (17), OTC −78 (78), others-1 (1)		Hosp.- 2 (4), lab.- 46 (92), OTC-1 (2), others-1 (2)			

Adm, admitted; Congest, congestion; Diff, difficulty; Discol, discoloured; Hosp, hospital; Lab, laboratory; na, not applicable; OTC, over the counter; PPE, personal protective equipment; yr, year.

The association between the subjects' occupation and health complaints was statistically significant (*P*=0.000), as 95% of waste handlers reported health complaints against 4% of control subjects.

The various specific complaints that had a statistically significant relationship with the occupation of waste handlers and control subjects, with waste handlers having more predilection to the specific complaint, are as follows: cough (*P*=0.000) 70 and 2%, difficulty breathing (*P*=0.000) 48 and 0%, chest pain (*P*=0.000) 26 and 0%, nasal congestion (*P*=0.000) 80 and 4%, runny nose (*P*=0.000) 61 and 0%, itchy eyes (*P*=0.000) 34 and 0%, skin rashes (*P*=0.000) 72 and 0%, itchy skin (*P*=0.000) 87 and 0%, discoloured skin (*P*=0.000) 95 and 2% and thickened skin (*P*=0.000) 92 and 0% among the waste handlers and control subjects, respectively.

Of all the specific health complaints, only having a bloody cough had an insignificant relationship with occupation (*P*=0.478) and was the least reported (1%) amongst the waste handlers.

On health-seeking behaviour and admission to hospitals, the factors of ever being admitted to hospital and admission to hospital in the past year had insignificant relationships (*P*=0.077 and 0.152), with 6 and 4% among the waste handlers, respectively, and 0% among the control subjects.

A statistically significant association was observed between respondents’ occupation and having laboratory tests (*P*=0.009), with waste handlers (26%) being three times more likely to access laboratory services compared to control subjects (8%).

On the availability and use of PPE, only 15% of the waste handlers had PPE gadgets, and only 3% of the number used them despite having them.

On the source of treatment for health complaints, the majority (78%) of the waste handlers obtained treatment through over-the-counter (OTC), 17% from laboratories, only 4% visited hospitals and 1% from other sources; conversely, among the control subjects, the majority (92%) accessed treatment from laboratories, 4% from hospitals and 2% each from OTCs and other sources, respectively.

### Frequency of bacteria isolates from the respiratory tracts and skin of the study subjects

A total of 704 bacterial isolates, which cut across 10 genera and 19 bacterial species, of which 11 are Gram-positive and 8 are Gram-negative, were obtained from the respiratory tract and skin of both the waste handlers and control subjects. Among the waste handlers, the percentage distribution of bacteria was almost the same at both sites: respiratory tract, 241 (49.9%) and skin, 242 (50.1%), compared to the control subjects’ respiratory tract, 105 (47.5%) and skin, 116 (52.5%), which showed a slight difference between the sites. *S. epidermidis* (24%) and *B. cereus* (13%) were more predominant in the respiratory tract, whereas *S. aureus* (29%) *and S. epidermidis* (19.0%) dominated the skin of the waste handlers. Similarly, *S. aureus* (34.3%) and *B. cereus* (20.9%) were more frequently detected in the respiratory tract, while *B. cereus* (37.1%) and *S. epidermidis* (18.1%) were detected more on the skin of the control subjects. Overall, among the waste handlers, *S. epidermidis* (21.9%), *S. aureus* (19.0%) and * B. cereus* (11.3%) had the highest pooled prevalence rates, as against *B. cereus* (29.4%), *S. aureus* (23.2%) and *S. epidermidis* (12.4%) among the control subjects. Details are shown in Table 3.

**Table 3.** Frequency of bacteria from the respiratory tract and skin of the study subjects (*n*=704)

**SN**	**Isolates**	**Waste handlers**					**Control subjects**				
		**Respiratory**		**Skin**			**Respiratory**		**Skin**		
		**Frequency**	**%**	**Frequency**	**%**	**Pooledprevalence (%**)	**Frequency**	**%**	**Frequency**	**%**	**Pooledprevalence(%)**
1	*Bacillus cereus*	33	13.6	21	9.0	11.3	22	20.9	44	37.9	29.4
2	*Bacillus subtilis*	11	4.5	30	12.3	8.4	6	5.7	6	5.2	5.45
3	*Enteroccocus faecalis*	11	4.5	4	2.0	3.2	–	–	–	–	–
4	*Enterococcus faecium*	–	–	1	0.4	0.2	–	–	–	–	–
5	*Escherichia coli*	10	4.1	11	4.5	4.3	12	11.4	3	3.0	7.2
6	*Klebsiella aerogenes*	5	2.0	14	6.0	4.0	–	–	–	–	–
7	*Klebsiella pneumoniae*	3	1.2	1	0.4	0.8	–	–	–	–	–
8	*Klebsiella oxytoca*	–	–	1	0.4	0.2	–	–	1	0.9	0.45
9	*Micrococcus luteus*	23	10.0	3	1.2	5.6	4	3.8	5	4.3	4.05
10	*Proteus mirabilis*	1	0.4	–	–	0.2	–	–	–	–	–
11	*Proteus vulgaris*	1	0.4	3	1.2	0.8	–	–	–	–	–
12	*Pseudomonas aeruginosa*	10	4.1	7	3.0	3.6	3	2.9	3	3.0	2.95
13	*Salmonella typhimurium*	9	3.8	5	2.1	2.95	–	–	–	–	–
14	*Staphylococcus aureus*	22	9.0	70	29	19.0	36	34.3	14	12.1	23.2
15	*S. epidermidis*	60	24.8	47	19.0	21.9	7	6.7	21	18.1	12.4
16	*Staphylococcus roseus*	12	5.0	11	4.5	4.75	1	1.0	–	–	–
17	*Staphylococcus xylosus*	10	4.0	10	4.1	4.05	5	4.8	13	11.2	8.0
18	*S. pneumoniae*	9	4.0	–	–	2.0	1	1.0	–		0.5
19	*Streptococcus pyogenes*	11	5.0	3	1.2	3.1	8	7.6	5	4.3	5.95
Total		241	100	242	100	100	105	100	116	100	100

### Frequency of fungi in the respiratory tracts and skin of the study subjects

A total of 191 fungal organisms were isolated from the respiratory tract and skin of both the waste handlers and the control subjects, and they cut across 6 genera and 10 fungi species. Among the study subjects, the percentage distribution of fungi showed marked differences between the sites: respiratory tract, 59 (41.3%) and skin, 84 (58.7%) of the waste handlers, as well as respiratory tract, 20 (41.7%) and skin, 28 (58.3%) for the control subjects. *Candida* was the dominant species among the study subjects. In the waste handlers, 81.4% were obtained from the respiratory tract and 42.9% from the skin. Similarly, among the control subjects, 85% were from the respiratory tract, while 78.5% were from the skin. Overall, *Candida* spp. had a pooled prevalence of 62.2 and 81.8% among the waste handlers and control subjects, respectively. This was followed by *Trichophyton* spp. (10%) in the waste handlers and *Cryptococcus* spp. (7.5%) in the control subjects. Details are shown in Table 4, Frequency of fungi in the respiratory tracts and skin of the study subjects

**Table 4.** Frequency of fungi genera among the study subjects (*n*=191)

**SN**	**Isolates**	**Waste handlers**					**Control subjects**				
		**Respiratory**		**Skin**			**Respiratory**		**Skin**		
		**Freq.**	**Percent**	**Freq.**	**Percent**	**Pooled Prev. (%)**	**Freq.**	**Percent**	**Freq.**	**Percent**	**Pooled Prev. (%)**
1	Alternaria *spp*.	1	1.7	9	10.7	6.2	–		–		0
2	Aspergillus *spp*.	3	5	3	3.5	4.3	–		3	10.8	5.4
3	Candida *spp*.	48	81.4	36	42.9	62.2	17	85	22	78.5	81.8
4	Cryptococcus *spp*.	4	6.8	7	8.3	7.5	3	15	–		7.5
5	Microsporum *spp*.	1	1.7	15	17.9	9.8	–		–		0
6	Trichophyton *spp*.	2	3.4	14	16.7	10.0	–		3	10.7	5.3
Total		59	100	84	100	100	20	100	28	100	100

## Discussion

### Sociodemographic characteristics of the study subjects

The findings of this study show that municipal waste handling in Aba is predominantly (95%) a male-dominated vocation. This finding is congruous with reports from other parts of Nigeria [22,23]. Sawyerr *et al.* [24] reported 61.7% male predominance in the waste management work in Ilorin Kwara State. Elsewhere, the studies by Vimercati *et al.* [2] in Italy (100% male) and Athanasiou *et al.* [3] in Greece (68% male) agree with our findings. On the other hand, the scenario is different in some climes, with differing results from our findings, where females were more involved in waste handling work than males [3,25, 26]. The predominance of males in waste handling work could be explained by the point of view of gender roles that waste handling work is tedious and strenuous, requiring excessive hard work and lifting of heavy bins, which requires masculine strength. For females, it may be explained by socioeconomic backwardness and low educational status.

This study shows the mean age of the waste handlers to be 30 years, and the age groups of 21–30 and 31–40 years were the groups with the highest proportions of 46 and 19%, respectively. This finding agrees with the findings of [24,25], who reported a mean age of 32 years.

Conversely, our findings differ in the mean age of respondents with the reports of other researchers, who observed a higher mean age range from 42 years to 53 years [2,3, 26].

### Bacterial distribution among study subjects

In terms of the presence of bacteria in the respiratory tract and skin of the study subjects, we isolated almost two and a half times more different bacterial species from the waste handlers compared to the control subjects, using the conventional culture techniques employed in this study (Table 3). Our finding agrees with the findings of other researchers who isolated some bacterial organisms from the bodies of waste workers [15,23, 27,30]. Similarly, several studies have reported the isolation of some of these organisms from solid waste, solid waste handling environments and dumpsites [10,22, 31,33]. This shows that waste handlers are prone to having more bacteria on them as a result of occupational exposure through direct contact with waste and via inhalation of organic dust laden with biological agents. These bacteria and some toxic bioproducts they elaborate, to which waste handlers might be exposed, are shown in Table 5.

**Table 5.** Pathogenicity-associated toxins and bio-products of the isolated bacteria

Bacteria	Toxins and pathogenicity bio-products	Pathologic role
*B. cereus*	Haemolytic enterotoxin haemolysis BL (HBL), non-haemolytic enterotoxin (Nhe), cytotoxin K (CytK) [47]	Diarrhoeagenic, haemolytic and necrotic intestinal syndromes
*B. subtilis*	Subtilisin (low toxigenic property) [48]	None
*Enteroccocus faecalis*	Cytolysin (pore-forming exotoxin) [49]	Increased toxicity/lethality in bacteraemia
*Enterococcus faecium*	Hydrogen peroxide [50]	Cell lysis, colonisation and infection
*Escherichia coli*	Shiga toxin [51]	Haemorrhagic colitis (HC), haemolytic uraemic syndrome (HUS)
*Klebsiella aerogenes*	Yersiniabactin (siderophore) and colibactin (genotoxin) [52]	Increased virulence and antibiotic resistance; carcinogenic potential
*Klebsiella pneumoniae*	Colibactin and a host of other pathologic bioproducts [53]	Hypervirulence and carcinogenic potential
*Klebsiella oxytoca*	Tilivalline and tilimycin [54]	Antibiotic-associated haemorrhagic colitis
*Micrococcus luteus*	Tilivalline and teichuronic acids [55]	Immune activation and systemic inflammation
*P. mirabilis* and *P. vulgaris*	HpmA and HlyA (cytotoxins and haemolysins) [56,57]	Induction of inflammation and lysis of cells/tissues in urologic conditions
*Pseudomonas aeruginosa*	Exotoxin A, leukocidin and a plethora of cytotins [58,59]	Apoptosis, pyroptosis and necroptosis of different organs and tissues
*Staphylococcus aureus*	Superantigen toxins (Sag toxins), membrane-damaged toxins (MDTs), exfoliative toxins (ETs) [60]	Hypervirulence, cytolysis, and immune modulation
*Staphylococcus epidermidis*	*N*-formylated alpha-helical peptide δ-toxin [61]	Biofilm formation and cytolysis
*Staphylococcus xylosus*	Superantigen toxins (Sag toxins) [62]	Immune activation/modulation
*Streptococcus pneumoniae*	Pneumolysin [63]	Cytotoxic/cytolytic and apoptosis of neuronal cells
*Streptococcus pyogenes*	Pyrogenic toxin superantigens (PTSAgs): streptococcal pyrogenic exotoxins (SPE A, C and J) [64]	Hypervirulence of STSS

STSS, streptococcal toxic shock syndrome.

### Fungal distribution among study subjects

We isolated fungi like *Alternaria* spp.*, Aspergillus flavus, Aspergillus fumigatus, Aspergillus lentulus, Candida* spp.*, Cryptococcus neoformans, Microsporum ferrugineum, Microsporum* spp.*, Trichophyton mentagrophytes* and *Trichophyton* spp. The findings of this study are consistent with the reports of [15,34], who, in addition to other fungi, isolated *Aspergillus* species and *Candida* species.

Similarly, it also partly agrees with the findings of some authors who isolated only *Aspergillus fumigatus* as the fungi species of interest [10,28, 35, 36]. Our findings show that *Candida*, *Trichophyton* and *Cryptococcus* are the most dominant genera among waste workers. This differs with the reports of other researchers who reported *Penicillium*, *Aspergillus* and *Cladosporum* as the dominant fungal genera [36,41]. This discordance could be explained by the differences in the sample types of the studies; as ours focused on microbial carriage on the bodies of waste workers, while the other studies focused on waste directly. Again, *Candida* species are recognised as normal flora and opportunistic pathogens that are plentiful on the skin and oropharyngeal regions of humans.

The presence of *Cryptococcus* species on the bodies of both the waste workers and the controls might be explained by the high environmental contamination from indiscriminate dumping of refuse of various origins prevalent in the Aba urban environment. This is in agreement with the study of [42], which recorded a 21.4% prevalence rate of *Cryptococcus* spp. from dust in some Brazilian libraries, and that of [11], which reported the presence of *Cryptococcus* species in MSW-related environments in Poland, and with reports elsewhere on the presence of the fungus in the urban environment [43,44].

In this study, *Microsporum* spp. are the third most frequently isolated fungi, with a frequency percentage of 9.8% and a higher preference for the skin amongst waste handlers. This finding might be explained by the fact that *Microsporum* is a multi-ecological niche-based organism; the waste handler encounters them in the course of his work. Again, the low rate of use of PPE by the waste handlers brings about direct contact of the body with the waste, which contains consortia of microorganisms. This finding agrees with the reports of other studies that isolated *Microsporum nanum* from air samples of landfill sites in Taiwan and *Microsporum canis* from hospital dumpsites in Aba, respectively [32,45].

This study recorded *Trichophyton* spp. as the second most frequently isolated fungus after *Candida* spp., with a frequency percentage of 10.0% and a higher preference for the skin amongst the waste handlers. This finding might be explained by *Trichophyton* spp. being a multi-ecological niche-based organism that the waste handler encounters in the course of his work and possibly the consequence of their low rate of use of PPE. This finding is in agreement with a study that isolated *Trichophyton* spp. (8.89%) from a composting facility using the Windrow method [46].

## Conclusion

This study highlighted that artisanal municipal solid waste handling in Aba is predominantly a male-dominated vocation, with the age group of 21–40 years and mean age of 30 years being in the majority. The waste handlers were mostly single, with secondary school as their highest level of education. Waste handling was significantly associated with some health complaints when compared to the control subjects in this study.

The prevalence of all-cause respiratory, eye, and skin complaints was higher among the waste handlers than the control subjects, which is an indication of an occupationally related challenge. This is evidenced by the fact that the waste handlers’ occupation exposed them to diverse microorganisms and health-affecting agents that negatively impacted their health. Furthermore, the observed low rate of possession and use of PPE amongst the waste handlers during their work is a strong indication of exposure to microorganisms and other deleterious substances that have been recognised to adversely affect the respiratory, eye, skin and overall health of the waste handlers.

This study also showed that different bacteria and fungi were more frequently detected in the respiratory tract and skin of waste handlers. The presence of the isolated bacteria and fungi in increased proportions in the waste handlers may be attributed to occupational exposure through direct contact with waste and via inhalation of organic dust laden with biological agents and poor working conditions of the waste handlers.

## Abbreviations

MSW: municipal solid waste
OTC: over-the-counter
PPE: personal protective equipment
